# The Laboratory Characterization of Fugitive Aerosol Emissions From a Standard Jet Nebulizer With and Without a Filtered Mouthpiece

**DOI:** 10.7759/cureus.50611

**Published:** 2023-12-15

**Authors:** Manhar Dhanak, Siddhartha Verma, Patrick G Hughes, Ai Ling Ching, Arthur Lo, Candice Clay, Adriana McKinney, John Frankenfield

**Affiliations:** 1 Department of Ocean and Mechanical Engineering, Florida Atlantic University, Boca Raton, USA; 2 Emergency Medicine, Florida Atlantic University, Boca Raton, USA; 3 Medical Affairs, Theravance Biopharma US, Inc., South San Francisco, USA

**Keywords:** simulation, mouthpiece filter, covid-19, healthcare worker safety, medical aerosols, aerosol-generating procedures

## Abstract

Background and objective

The risk of severe acute respiratory syndrome coronavirus 2 (SARS-CoV-2) transmission from patients with coronavirus disease 2019 (COVID-19) during nebulization is unclear. In this study, we aimed to address this issue.

Methods

Fugitive emissions of aerosolized saline during nebulization were observed using a standard jet nebulizer fitted with unfiltered and filtered mouthpieces connected via a mannequin to a breathing simulator. Fugitive emissions were observed by using a laser sheet and captured on high-definition video, and they were measured by using optical particle counters positioned where a potential caregiver may be administering nebulization and three other locations in the sagittal plane at various distances downstream of the mannequin.

Results

The use of a standard unfiltered mouthpiece resulted in significant emission of fugitive aerosols ahead of and above the mannequin (spread over 2 m in front). A mouthpiece with a filter-adaptor effectively suppressed the emissions, with only minor leakage from the nebulizer cup. Particle count measurements supported the visual observations, providing total particle count levels and aerosol concentration levels at the measurement locations. The levels decayed slowly with downstream distance.

Conclusions

The visualization described above captured the dispersion of emitted aerosols in the plane of the laser sheet, aligned with the sagittal plane. The particle count measurements provided temporal and spatial distributions of the aerosol concentration levels over the time and locations considered. However, the exhaled air and aerosolized droplets spread three-dimensionally in front of and above the mannequin. The results visually highlight the effectiveness of using a filtered mouthpiece in suppressing the fugitive aerosols and identify an approach for limiting the occupational exposure of healthcare workers to these emissions while administering nebulized therapies.

## Introduction

Minimizing viral transmission during aerosol-generating procedures (AGPs) has become critical to healthcare worker safety during the coronavirus disease 2019 (COVID-19) pandemic. Therapeutic respiratory medication can be administered via a variety of nebulizers that generate aerosolized droplets of the medicine. Fugitive emissions of these medical aerosols, as well as bioaerosols generated in respiratory exhalations, can travel significant distances from the source and remain in the air for several minutes, depending on multiple environmental factors (temperature, humidity, airflow, etc.) [1]. These aerosols, therefore, pose the risk of secondary exposure to bystanders and healthcare workers [2]. Furthermore, the administration of nebulization therapy to patients with respiratory diseases may expose healthcare workers to fugitive aerosols that could potentially contain pathogens.

There are transmission risks associated with such exposure, particularly during the COVID-19 pandemic. Tran et al. [3] cite studies related to the first severe acute respiratory syndrome coronavirus (SARS-CoV) outbreak, two of which reported nosocomial transmission potentially related to nebulization and one that showed otherwise. Transmission was attributed to prolonged exposure to infected individuals and a lack of infection control measures. A more recent review by Goldstein et al. [1] that included SARS-CoV-2 and non-coronavirus (influenza) studies suggests that the risks could not be ruled out given such inconclusive evidence. The World Health Organization (WHO) [4,5] and Centers for Disease Control and Prevention (CDC) [6] recommend that healthcare workers wear appropriate personal protective equipment (PPE) such as a respirator, eye protection, gown, and gloves when conducting AGPs on patients with respiratory infections [3]. In the setting of providing nebulized therapies to COVID-19-infected people, several publications have provided guidance and practical strategies over the last two years [7-12]. They mostly advocate for appropriate consideration for the inhaled medication delivery method, including the type of nebulizer (standard jet, vibrating mesh, and breath-actuated devices) and breathing interfaces (mouthpiece versus a facemask) [7,8,13-15].

In this study, we examine, through visual observation and particle count measurement, in a laboratory simulation, the emission of fugitive aerosols from a jet nebulizer and their attenuation through the use of a filtered mouthpiece. A previous study, involving visualization of airflow during nebulization of sterile water, has been reported by Hui et al. [16]. In that study, leakage of exhaled air through the side vents of a jet nebulizer connected to a human patient simulator (HPS) was visualized using tracer smoke particles that were continuously introduced into the HPS lung as part of the inhaled air. In other studies, scholars have significantly decreased particle generation during nebulizer therapy by adding a viral filter [17,18]. Here we provide visual observations, backed by particle count measurements, of the results, including both temporal and spatial variations of the concentrations of the fugitive aerosols for unfiltered and filtered jet nebulizer mouthpieces. In contrast, in the present study, fugitive emissions of aerosolized droplets of nebulized saline, representative of therapeutic medical aerosols, are directly visualized and concentration levels of the fugitive aerosols in the vicinity of and at various distances from the emission source are measured using a set of particle counters. The impact of fitting the nebulizer with a filter on the fugitive emissions is examined. The observations are exclusive of bioaerosols that the patient may have generated during exhalation.

## Materials and methods

**Figure 1:**
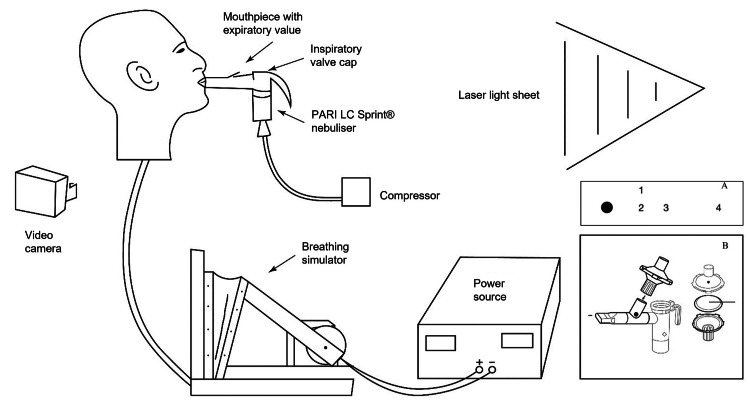
Schematics of the test setup (not to scale) Inset A shows the position of the four optical particle counter measurement stations (numbered 1–4) relative to the mannequin (large black circle). Inset B shows the PARI Filter and Valve Set for the LC Nebulizer (manufacturer’s schematics)

A schematic illustration of the experimental setup is shown in Figure 1. A mannequin manufactured by Only Mannequins (Model: 50013) attached to a custom bellows-driven breathing simulator was used to represent an adult patient per United States Pharmacopeia Chapter <1601> (Products for Nebulization). The bellows-based simulator was custom-built and calibrated in the College of Engineering at Florida Atlantic University. An electrically controlled motor drives a 500 mL volume bellows chamber to simulate the breathing. The power supply to the simulator was calibrated to provide the required breathing cycle rates. A simulated breathing rate of 15 breaths per minute with a tidal volume of 500 mL and an inspiratory-to-expiratory ratio of 1:1 was used. A standard jet nebulizer (PARI LC Sprint) was attached to the mannequin by using a mouthpiece interface. The nebulizer was driven by a compressor (PARI Trek S) delivering airflow at 4 L/min to the nebulizer reservoir, which was filled with normal saline [0.90% weight per volume (w/v) of NaCl]. The breathing simulator did not draw air from any source other than the nebulizer mouthpiece during inhalation; this was ensured by tightly sealing the mouthpiece with tape. Saline aerosols were drawn into the mannequin via the mouthpiece interface during the inhalation phase of the breathing cycle and emitted through the expiratory valve port on the mouthpiece during the exhalation phase. The emitted aerosols were visualized in a thin sheet of green laser light (532 nm wavelength) positioned in front of the mannequin, with the plane of the sheet coincident with the sagittal plane, and the observations were captured using a high-definition video camera. The camera was placed so that its view was either normal to the light sheet or at an angle so that it faced the laser light source. The latter placement allowed optimization of the imagery since the droplets scatter a significant portion of the light away from the source, as well as facilitate visualization of the emissions to a further distance away from the mannequin.

The visual observations were complemented by particle count measurements using N3 optical particle counters (OPCs) from Alphasense Ltd. One OPC was placed at Station 1, located 40 cm ahead and 30 cm to the side of the mannequin, corresponding to where a possible caregiver administering the nebulization may be positioned. OPCs were also placed on tripods located at three observation stations in the sagittal plane downstream of the mannequin as indicated in Figure 1 (Inset A). Stations 2-4 were located 40, 80, and 160 cm respectively from the mannequin. These parameters were selected to characterize the spread of the aerosols at a distance away from the source. The measurements were made simultaneously and synchronized using a single computer for data acquisition from the four sensors. Each OPC recorded particle counts in 24 bins of diameter sizes d*_i_ *(*i *= 1 to 24), ranging from 0.4-40 micrometers over one-second time intervals using sampling flow rates of Q_s_ = 4.7 x 10^-6^ m^3^/s.

Three sets of tests were conducted with the jet nebulizer, one using a standard unfiltered mouthpiece with an expiratory valve port (Figure 1) and the other with a mouthpiece fitted with and without an exhalation filter-adaptor (PARI Filter and Valve Set for LC Nebulizer; Figure 1, inset) [18]. The study was carried out over 2021-2022. In each case, 3 mL of saline was nebulized (15-20 minutes). Control runs of the compressor and the breath simulator were conducted in the absence of saline before and after nebulization to detect any spurious aerosols present in the system. The nebulization runs were replicated twice.

## Results

**Figure 2:**
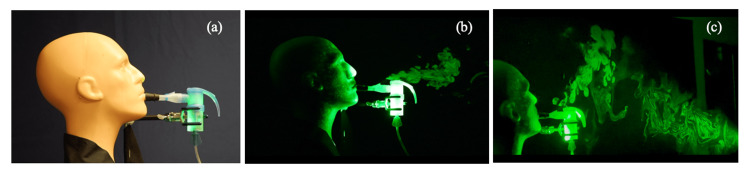
Test setup without a filter and observed aerosol emissions (a) Test setup with a standard PARI mouthpiece without a filter; (b) and (c) observed emission of aerosols from the jet nebulizer during exhalation

**Figure 3:**
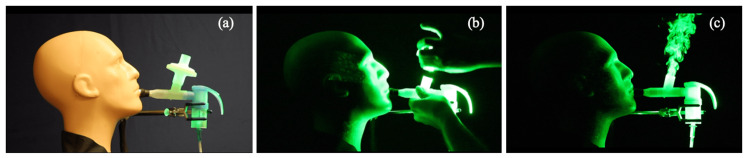
Test setup with a filter, removal of the filter, and observed aerosol emissions (a) Test setup with a PARI filter set; (b) removal of the filter piece; (c) observed emission of aerosols in the absence of the filter during exhalation

**Figure 4:**
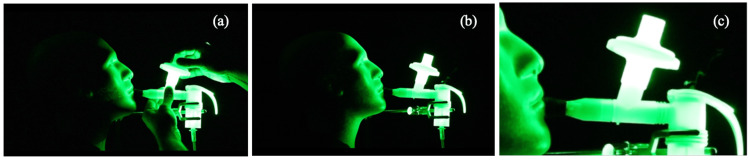
Replacement of the filter and observed aerosol emissions (a) Replacement of the PARI filter piece; (b) observed exhalation cycle showing the absence of the emission of fugitive aerosols from the expiratory valve; (c) expanded view of Figure 4b to illustrate minor leakage from the nebulizer cup

The results of the visualization of the fugitive aerosol emissions are shown in Figures 2-4. Figures 2a-2c show the results of the first set of tests using the standard mouthpiece with the expiratory valve port. At each exhalation, a jet of aerosolized droplets was emitted from the exhalation valve on the mouthpiece. The shape of the exhalation valve directed the jet upwards over the mouthpiece. The momentum of the jet then resulted in a three-dimensional spread in the form of a turbulent puff, carrying the fugitive aerosols ahead and above the mannequin, initially extending 0.6 to 0.8 m from the mannequin (Figure 2b). However, the aerosolized droplets, given their small size and relatively large surface area compared to their weight, persisted in the air for several minutes and were convected further away from the mannequin in the ensuing airflow accompanying the spread of the droplets in the room. The ambient temperature and relative humidity in the room were 22 ^o^C and 47%, respectively, with minimal air exchange. Over the 15- to 20-minute period of operation, the fugitive aerosols emitted in the exhaled air were observed to spread into the open space ahead and above the mannequin, extending to over 2 m in front of it. Figure 2c depicts the view looking toward the light source that enables the best observation of the dispersion of the aerosolized droplets in the light sheet.

The results of the second set of tests using the mouthpiece fitted with the PARI filter-adaptor are shown in Figures 3-4. When the filter-adaptor was removed (Figure 3b), the aerosolized droplets were emitted in a jet of air during each exhalation as before, but with the jet directed at a steeper upward angle by the shape of the expiratory port (Figure 3c). When the filter-adaptor was connected to the expiratory port (Figure 4a), fugitive aerosols were no longer observed (Figure 4b). The filter-adaptor captured aerosols in the exhaled breath and significantly reduced the emission of fugitive aerosols during nebulization. Only minor emissions were observed over the inspiratory valve cap (Figure 4c).

**Figure 5:**
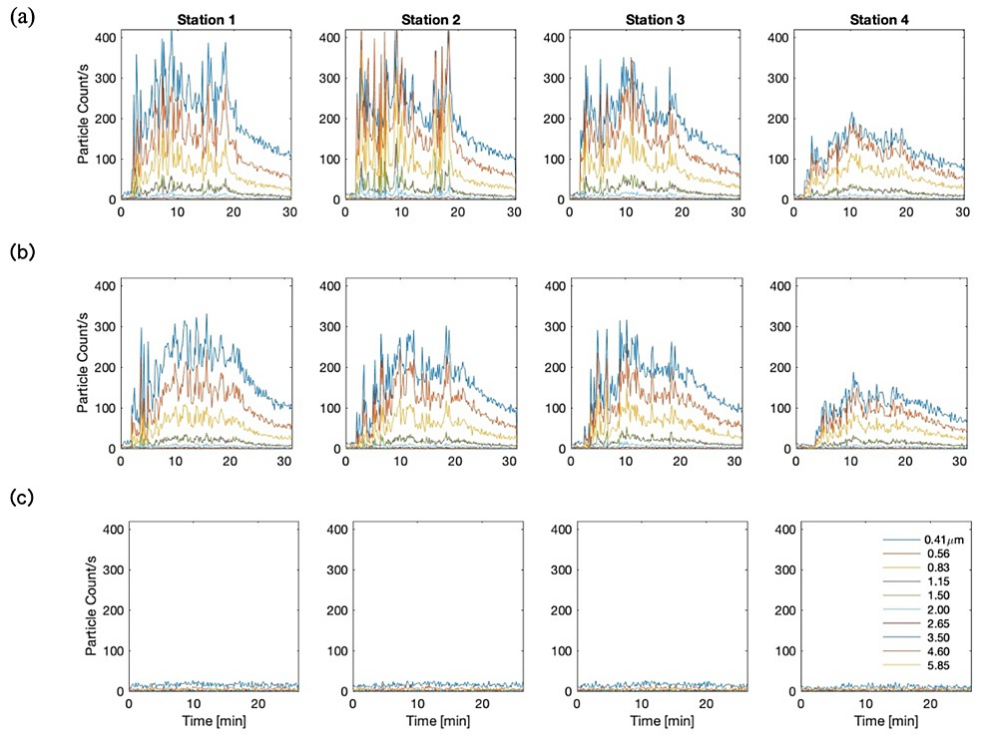
Time series of particle count measurements at the four OPC measurement stations (a) Standard PARI mouthpiece without a filter; (b) PARI mouthpiece with a filter set but with the filter cap absent; (c) PARI mouthpiece with a filter set, with the filter cap properly in place OPC: optical particle counter

**Figure 6:**
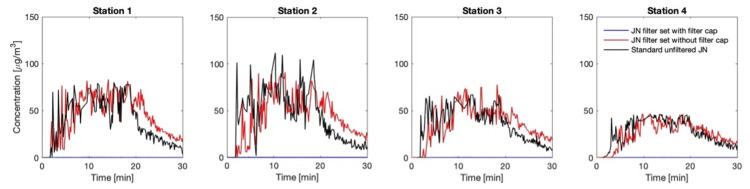
Temporal and spatial variation in aerosol concentration Measured concentration at the four OPC stations for each of the cases a–c defined in Figure 5 OPC: optical particle counter

**Figure 7:**
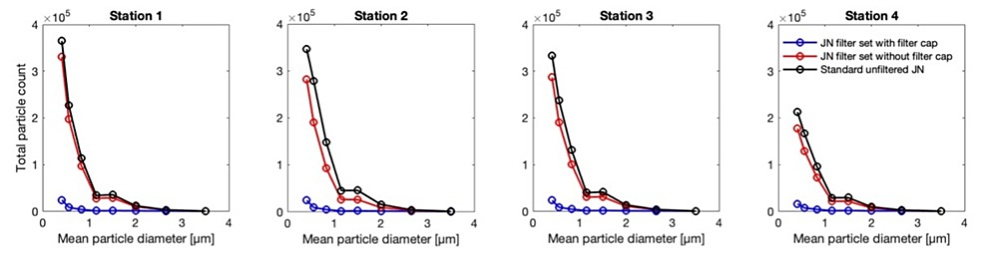
Total particle count at the four OPC measurement stations as a function of particle size Measured concentration at the four OPC stations for each of the cases a–c defined in Figure 5 OPC: optical particle counter

The results of the aerosol particle count measurements using the OPCs are presented in Figures 5-7. Figure 5 depicts the time series of the measured particle counts for a range of particle sizes at each of the four observation stations for the cases of (a) a regular jet nebulizer without a filter, (b) a jet nebulizer equipped with a PARI filter but with the filter cap removed, and (c) a jet nebulizer with the PARI filter, including the filter cap. The time series have been lowpass filtered at 1/40 Hz. As can be seen, in the absence of the filter, the fugitive aerosols spread into the open space, the aerosol levels decaying with distance from the emission source, albeit slowly, as the plume expands and engulfs ambient air. The observations at Stations 1 and 2 are similar, highlighting the three-dimensional spread of the fugitive aerosols. Furthermore, the spread of the aerosols in the unfiltered cases (a) and (b) show similar patterns. Decay of the particle count with time beyond 20 minutes represents observations following sputter. The particle counts in the filtered jet nebulizer case (c) dramatically illustrate the effectiveness of the PARI filter in filtering out the aerosols, with the particle count measurement at the observation stations corresponding to ambient levels over the entire period of nebulization. 

Comparisons of the associated aerosol concentrations between cases a-c at the four stations are provided in Figure 6. The figure further illustrates the effectiveness of the filter and the features described above. The concentrations are based on taking into account the aggregate volume of the aerosol particles, regarding them to be spherical. Between cases (a) and (b), the aerosol spread associated with (a) appears to have greater variability with significantly higher peaks in particle count, presumably since the direction of the emitted aerosol from the standard mouthpiece (Figure 2) is directly towards the OPCs, whereas in the case of the mouthpiece without the filter cap (Figure 3c) is upwards. Finally, the total particle count as a function of particle size for cases (a)-(c) is compared at each station in Figure 7. As may be expected, the total count of the smallest aerosols dominates, and the total count drops off with an increase in the size of the aerosols. Furthermore, the counts reduce with the distance from the source of emission.

## Discussion

The present study involves a specific (PARI) jet nebulizer with and without the placement of a filter-adaptor on the expiratory valve of its mouthpiece. The study was limited to the consideration of the dispersion of aerosolized droplets of normal saline generated as part of nebulization so that it can be considered to represent medical aerosols. The lack of movement from the simulator might impact the aerosols' distribution and the mask's fitting. Bioaerosols generated within the patient’s respiratory system were not considered. The visualization described here captures the dispersion of the fugitive emissions in the plane of the laser sheet, aligned with the sagittal plane. Corresponding particle count measurements provide temporal and spatial distributions of the aerosol concentration levels over the time and locations considered. However, the exhaled jet of air and aerosolized droplets spread three-dimensionally in front of and above the mannequin. Visual observation and aerosol count measurement of such a spread can be facilitated by using multiple laser sheets and several particle counters, respectively. The method of visualization can be utilized to characterize fugitive emissions of medical aerosols from other nebulizers and to evaluate the effectiveness of other types of fugitive emission mitigation devices [15,16].

The results of the flow visualization and particle count measurement show that aerosolized droplets leak significantly from the expiratory valve of a standard mouthpiece of a jet nebulizer during simulated exhalation. The droplets spread in front of and above the mannequin, remaining suspended in the air for minutes and stretching to over 2 m from the mannequin over the 15- to 20-minute period of the nebulization operation. Replacing the standard jet nebulizer mouthpiece with one that includes a PARI filter-adaptor suppresses this leakage; only low levels of fugitive emissions from the inspiratory valve cap on the nebulizer are apparent near the cap. In this case, particle count measurements, unlike the standard mouthpiece, show that the associated fugitive aerosol concentration levels are not elevated above the ambient level. Thus, our findings show that a filter-adaptor on the expiratory valve of the mouthpiece of a jet nebulizer is effective in suppressing emissions of fugitive aerosols from the valve during nebulization. The addition of a filtered mouthpiece may decrease the risk of secondary exposure to bystanders and healthcare workers to respiratory pathogens. Future areas of study include the effects of room ventilation, including negative and positive airflows. In addition, the study can be repeated to characterize the spread of fugitive aerosols for other types of nebulizers. Here, we provide visual observations, backed by particle count measurements, of the results, including both temporal and spatial variations of the concentrations of the fugitive aerosols for unfiltered and filtered jet nebulizer mouthpieces.

## Conclusions

Our study adds to the evidence of the risks that healthcare workers face from exposure to respiratory pathogens while administering nebulized therapies. The results visually highlight the effectiveness of using a filtered mouthpiece in suppressing the fugitive aerosols and identify an approach for limiting the occupational exposure of healthcare workers to these emissions.
